# HIV-1 molecular transmission networks among MSM in Ningxia, China (2018–2024): insights into local transmission dynamics and drug resistance

**DOI:** 10.3389/fmicb.2026.1766785

**Published:** 2026-06-08

**Authors:** Zhonglan Wu, Jianxin Pei, Ziyang Luo, Yufeng Li, Xiaohong Zhu, YouPing Duan, Subinuer Mutalifu, Yong Li

**Affiliations:** 1 College of Life Science, Ningxia University, Yinchuan, Ningxia, China; 2 Ningxia Center for Disease Control and Prevention, Yinchuan, Ningxia, China; 3 College of Public Health, Ningxia Medical University, Yinchuan, Ningxia, China

**Keywords:** China, drug resistance, HIV-1, molecular transmission network, MSM

## Abstract

**Objectives:**

Men who have sex with men (MSM) remain a high-risk group for HIV infection, accounting for nearly half of new HIV cases worldwide—a persistent global public health concern. This study investigated HIV-1 molecular transmission networks among MSM in Ningxia Hui Autonomous Region (NHAR), China. The aim was to characterize transmission dynamics within.

**Methods:**

Plasma samples were collected from HIV-positive MSM in Ningxia between 2018 and 2024. HIV-1 RNA was extracted and sequenced for genotyping and drug resistance analysis. Genetic distances were calculated using the TN93 model in HyPhy 2.2.4. A sensitivity analysis was performed across thresholds of 0.1%–2.1% to justify the chosen threshold of 1.5%. Molecular transmission networks were visualized in Cytoscape 3.7.0. Bayesian phylogenetic analysis was used to identify migration events. Multivariable logistic regression was used to assess factors associated with clustering and high linkage.

**Results:**

Among 296 MSM with successful sequencing, CRF07_BC was the predominant subtype (57.09%). CRF01_AE (20.61%) and CRF55_01B (5.07%) were also common. A total of 2.70% were classified as unique recombinant forms (URFs). We identified 129 MSM (43.6%, 129/296) within molecular transmission clusters. Large clusters (≥10 members) contained 117/129 (90.7%) of clustered individuals.CRF07_BC was the most frequent subtype in clusters (71.3% of clustered cases), but logistic regression showed it was associated with lower odds of high-degree connectivity (AOR = 0.209). Most clustered cases were from Yinchuan (57.4%). The strongest connection intensity appeared within Yinchuan, then between Yinchuan and Wuzhong. The most robust network connections were among MSM aged 25–39. Basic reproduction number (R_0_) analysis showed the CRF01_AE subtype remained stable. CRF07_BC showed an increasing trend since 2020, while other subtypes rose sharply after 2022. Employment as a worker (AOR = 4.019) and infection with CRF07_BC (AOR = 0.209) or CRF55_01B (AOR = 0.201) were significantly associated with high-degree linkage; age groups showed no statistically significant associations.

**Conclusion:**

MSM aged 25–39 showed the highest connectivity in transmission networks. The high clustering rate (43.6%) and identified acquired drug resistance (38.2% among sequenced individuals) underscore the urgent need for targeted interventions. These findings are limited to ART-experienced MSM with viremia (VL > 200 copies/mL) and cannot be generalized to the broader MSM population or to virologically suppressed individuals.

## Background

Acquired immunodeficiency syndrome (AIDS) is a chronic infectious disease caused by the human immunodeficiency virus (HIV). As of 2024, an estimated 39.9 million people worldwide were living with HIV (GBD 2021 HIV Collaborators, 2021). Among key affected populations, MSM represent a persistent global public health challenge. In recent years, HIV prevalence and incidence among MSM have remained high. In China, the proportion of newly reported HIV cases attributed to MSM increased from 2.5% in 2006 to 25.6% by 2022 (Han, 2023). The overall HIV prevalence among MSM in China is approximately 7%, with rates in some cities, particularly in the southwest, reaching as high as 12.1% (Wu et al., 2025; Zhang et al., 2016).

In response to these challenges, HIV molecular transmission network analysis has emerged as a powerful and widely recognized tool in HIV epidemiology. This technique uses genetic sequences from individuals living with HIV to infer potential transmission linkages and patterns (Zhang et al., 2022), serving as a valuable complement to traditional social and sexual network research (Dennis et al., 2014; Leigh Brown et al., 2011; Oster et al., 2015). The growing application of molecular network analysis offers new insights for HIV prevention and control efforts (Zhang et al., 2023). By identifying high-risk individuals and transmission clusters, this approach enables targeted public health interventions aimed at interrupting transmission chains. Furthermore, it facilitates the timely identification of undiagnosed individuals within risk networks and supports proactive prevention efforts among uninfected individuals, offering a precision-based strategy to reduce HIV incidence and improve the allocation of public health resources.

Although overall HIV prevalence remains lower than in southwestern China, recent surveillance data indicate a steady increase in both HIV incidence and detection rates among MSM, highlighting this group as a key driver of local transmission. Several risk factors contribute to this trend, including high rates of unprotected anal intercourse, multiple sexual partnerships, low consistent condom use, and limited access to tailored HIV prevention services. Additionally, socio-structural factors such as stigma, limited health literacy, and high mobility between Ningxia’s major cities, particularly between the capital Yinchuan and surrounding areas may further facilitate transmission networks. These emerging patterns underscore the need for a deeper understanding of the molecular transmission dynamics to guide targeted interventions in this previously understudied population (Liu et al., 2025; Pei et al., 2024).

This study aimed to characterize the HIV-1 transmission dynamics by investigating molecular transmission networks among MSM in the Ningxia Hui Autonomous Region (NHAR), China. The findings are intended to provide a scientific basis for developing targeted public health strategies to enhance HIV-1 control in this key population. However, it is important to note that this study focuses specifically on ART-experienced MSM with detectable viremia (viral load > 200 copies/mL), as these individuals are most relevant for identifying active transmission chains and acquired drug resistance. Findings may not be generalizable to the entire MSM population in Ningxia.

## Materials and methods

### Study participants

Participants were included in this study if they met the following criteria: (1) HIV-positive diagnosis confirmed via initial screening with an HIV-1/HIV-2 enzyme-linked immunosorbent assay (ELISA) and confirmed by HIV-1 Western blotting at the Ningxia Center for Disease Control and Prevention (CDC); (2) Reported sexual transmission as the sole route of infection, specifically identifying as MSM; (3) Had received antiretroviral therapy (ART) for more than six months; (4) Had a viral load exceeding 200 copies/mL; and (5) Provided verbal or written informed consent to participate in the study voluntarily. Thus, our study population represents a subgroup enriched for virologic failure or poor adherence, and the results are not generalizable to virally suppressed MSM or to treatment-naïve individuals.

Demographic information (ethnicity, sex, age, and marital status) was obtained from the Ningxia Information System for Disease Control and Prevention. After removing duplicate entries, pol gene sequences (covering 1,060 base pairs, HXB2: 2253–3313) were successfully obtained from 296 people living with HIV (PLWH) for analysis.

### Ethics statement

Ethical approval for this study was obtained from the Ethics Review Committee of the Ningxia Hui Autonomous Region Center for Disease Control and Prevention. A statement confirming that formal consent was obtained in writing from the Participants.

### Laboratory testing

Plasma was separated from whole blood and stored at −80 °C. HIV RNA was extracted from 200 μL of plasma using an automatic nucleic acid extractor (Zhuhai Lizhu Reagent Co. Ltd.), following the manufacturer’s protocol. The HIV-1 pol gene, including the complete protease region (codons 1–99) and the first 300 amino acids of the reverse transcriptase region (codons 1–300), was amplified using nested polymerase chain reaction (nPCR) with an in-house method. The amplified product was approximately 1.1 kb. Electrophoresis was performed on a 1% agarose gel, and positive products were purified and sequenced by Hailite Guangzhou Gene Sequencing Ltd.

### Sequence analysis

Raw sequences were edited and assembled using Sequencher 4.9, and aligned with BioEdit 7.1.3. A phylogenetic tree was constructed in MEGA 7.0 using the Neighbor-Joining method (Kimura two-parameter model, 1,000 bootstrap replicates). HIV-1 subtypes were determined by comparison with international reference strains from the HIV database (LANL^1^). Sequences that could not be subtype-confirmed by phylogenetic tree analysis or HIV BLAST were categorized as unique recombinant forms (URFs), and recombination breakpoints were further analyzed using SimPlot.

### Bayesian phylogenetic and systematic-dynamical analysis

To examine the transmission dynamics of different HIV-1 subtypes across age groups and geographic regions, a Bayesian phylogenetic framework was employed.

### Temporal signal assessment

Initial maximum likelihood phylogenetic trees were constructed in MEGA 12 and exported as Newick files. These were assessed for temporal signal using TempEst v1.5.3, based on the best-fitting root. Sequences with extreme values in scatter and residual plots were corrected or removed. A correlation coefficient (R^2^) > 0.3 was required to proceed.

### Bayesian phylogenetic analysis

Bayesian analyses were conducted in BEAST v1.10.5 using a Skygrid coalescent model under a general time-reversible (GTR) substitution model with an uncorrelated relaxed clock. Maximum clade credibility (MCC) trees were generated using BEAUti, with convergence evaluated by effective sample size (ESS) ≥ 200 in Tracer v1.7.2. Bayesian stochastic search variable selection (BSSVS) was used to identify subgroup relationships, and Markov jumps were applied to estimate the expected number of viral migrations. SpreaD3 v0.9.6 was used to calculate Bayes factors and posterior probabilities, with results filtered by strict thresholds (Bayes factors ≥ 3; posterior probabilities ≥ 0.8).

### Systematic-dynamical analysis

A Birth-Death Skyline model was implemented under a GTR substitution model with a relaxed log-normal molecular clock using BEAUti v2.7.7 to generate XML files. Analyses were performed in BEAST v2.7.7, and ESS ≥ 200 was verified in Tracer v1.7.2. Output log files were processed in R v4.4.0 using the bdskytools package to dynamically visualize temporal changes in the reproductive number (R_0_) and reconstruct epidemic trajectories.

### Drug resistance analysis

Nucleotide sequences were submitted to the Stanford University HIV Drug Resistance Database^2^ for mutation identification and antiretroviral drug susceptibility analysis. Drug resistance levels were classified into five categories based on mutation scores: susceptible (S), potential low-level resistance (P), low-level resistance (L), intermediate resistance (I), and high-level resistance (H). Any sequence exhibiting at least one low-to-high-level resistance mutation was defined as drug-resistant.

Given that all participants were ART-experienced with VL > 200 copies/mL, the detected resistance represents acquired drug resistance (ADR) rather than transmitted drug resistance (TDR).

### Genetic transmission network analysis

Pairwise genetic distances were calculated using the TN93 model in HyPhy v2.2.4. A genetic distance threshold of 0.015 (1.5%) was selected based on published literature (National Center for AIDS/STD Control Prevention and China CDC, 2021) and a sensitivity analysis. We tested thresholds of 0.1%–2.1%. The clustering rate and network topology remained stable across 0.9%–1.9% (see Supplementary Table 1 and Supplementary Figure 1), supporting the robustness of our choice. Molecular transmission networks were visualized using Cytoscape v3.7.2, where the number of edges connected to each node represented its “degree” value, indicating potential transmission linkages. Clusters were categorized as small (2–5 members), medium (6–9 members), or large (≥10 members). Nodes with degree ≥ the median (degree ≥ 3) were defined as “high-degree nodes.” The term “super-spreader” has been removed as it implies directionality and confirmed transmission, which our molecular network cannot establish.

To assess the potential impact of convergent evolution at drug-resistance mutation (DRM) sites, we performed a sensitivity analysis masking major DRM codons (PR: 30, 32, 33, 46, 48, 50, 54, 76, 82, 84, 90; RT: 41, 65, 67, 70, 100, 103, 106, 108, 151, 179, 181, 184, 190, 210, 215, 225, 230, 236, 238) and re-constructed the network. Clustering rates decreased by 3%–5%, indicating that DRM-driven spurious links exist but do not fundamentally alter our conclusions.

### Statistical analysis

A study database was constructed in Microsoft Excel 2010, and statistical analyses were conducted using SPSS v22.0. Categorical variables were summarized as counts and proportions. Differences between groups were assessed using the chi-square test or Fisher’s exact test, as appropriate. Logistic regression analysis was employed to identify factors associated with high-degree linkage (degree ≥ median). All statistical tests were two-tailed, and *P* < 0.05 was considered statistically significant.

## Results

### Study population

Between 2018 and 2024, a total of 855 individuals infected with HIV-1 who had received antiviral treatment within the MSM demographic were sampled. In 2024, 354 individuals were found to have a viral load exceeding 200 copies/mL, meeting the criteria for inclusion in the sequencing analysis. Among them, 83.6% (296/354) yielded successful pol gene sequences and were included in the final analysis. A flow diagram is provided in Supplementary Figure 2.

As illustrated in Table 1, the age distribution of the 296 sequenced participants was as follows: 25–39 years: 169 individuals; ≥40 years: 123 individuals; <20 years: 16 individuals.

**TABLE 1:** Socio-demographic characteristics of HIV-1 infected patients among men who have sex with men (MSM) in Ningxia.

Characteristics	Total (%)	2018	2019	2020	2021	2022	2023	2024	c^2^ value	*P-*value
Age, (*n*/%)									38.421	0.091
<20	16 (5.41%)	1	0	0	2	7	5	1	–	–
20∼29	93 (31.42%)	5	3	9	7	9	21	39	–	–
30∼39	89 (30.07%)	7	4	9	15	11	23	20	–	–
40∼49	60 (20.27%)	8	2	5	5	6	10	24	–	–
50∼59	29 (9.80%)	1	2	3	1	3	11	8	–	–
≥60	9 (3.04%)	0	0	0	0	2	4	3	–	–
Occupation, (*n*/%)									20.854	0.652
Unemployed/retiree	106 (35.81%)	10	1	6	12	14	29	34	–	–
Farmer	41 (13.85%)	2	3	5	4	3	10	14	–	–
Business waiter	39 (13.18%)	2	3	5	3	6	7	13	–	–
Workers	32 (10.81%)	1	3	1	1	7	10	9	–	–
Others and unknown	78 (26.35%)	7	1	9	10	8	18	25	–	–
Marital status (*n*/%)									16.913	0.135
Married/partnered	62 (20.95%)	5	4	9	5	6	15	18	–	–
Divorced/widowed	55 (18.58%)	8	4	4	5	9	12	13	–	–
Unmarried	179 (60.47%)	9	3	13	20	23	47	64	–	–
Degree of education									13.487	0.943
Illiterate	6 (2.03%)	0	1	0	1	0	1	3	–	–
Primary school	18 (6.08%)	0	1	2	1	2	4	8	–	–
Junior high school	71 (23.99%)	6	3	9	7	9	18	19	–	–
High school or technical secondary school	81 (27.36%)	5	3	7	10	12	22	22	–	–
College degree or above	120 (40.54%)	11	3	8	11	15	29	43	–	–
Drug resistance									19.884	0.003
Yes	113 (38.18%)	9	8	14	6	21	26	29	–	–
No	183 (61.82%)	13	3	12	24	17	48	66	–	–
HIV-1 genotype									32.898	0.047
CRF01_AE	61 (20.61%)	7	1	6	11	4	18	14	–	–
CRF07_BC	169 (57.09%)	13	7	14	16	26	31	62	–	–
CRF55_01B	15 (5.07%)	1	1	3	1	0	4	5	–	–
URFs	8 (2.70%)	0	1	0	0	1	2	4	–	–
Others	43 (14.53%)	1	1	3	2	7	19	10	–	–
Place of residence									22.558	0.463
Yinchuan city	179 (60.47%)	14	7	16	26	20	41	55	–	–
Shizuishan city	29 (9.80%)	0	1	2	2	4	8	12	–	–
Wuzhong city	53 (17.91%)	6	3	5	2	10	14	13	–	–
Guyuan	11 (3.71%)	1	1	1	1	2	2	3	–	–
Zhongwei	24 (8.11%)	1	0	2	1	2	7	11	–	–

The demographic composition of the population was as follows: 106 individuals (35.81%) were unemployed or retired, 41 (13.85%) were farmers, 39 (13.18%) were business people, and 32 (10.81%) were waiters, and 78 (26.35%) were classified as “other” or had missing occupational data. In terms of education, 40.54% (120/296) of participants held a college degree. Geographically, the majority of individuals 60.47% 179/296) were residents of Yinchuan, the capital of Ningxia.

### HIV-1 subtype distribution and drug resistance

**FIGURE 1:**
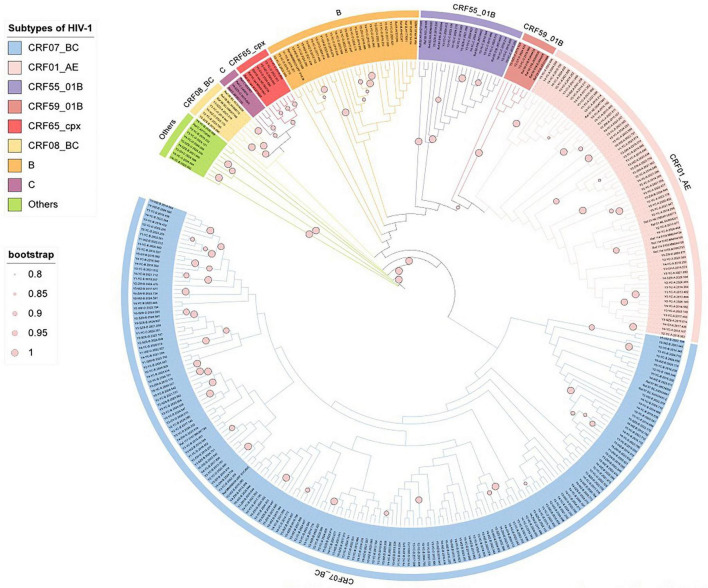
Neighbor-Joining phylogenetic tree of 296 pol sequences of HIV-1 in Ningxia. A total of 61 HIV-1 sequences were branched with the CRF01_AE reference sequence (depicted in pink), 169 HIV-1 sequences were branched with the CRF07_BC reference sequence (depicted in blue), 20 HIV-1 sequences were clustered with the B reference sequence (depicted in orange), and 15 HIV-1 CRF55_01B sequences were identified (depicted in purple), 3 CRF65_cpx sequences (depicted in red), 3 CRF58_01B sequence (depicted in light red), and 8 URFS (depicted in green) were also identified the reference sequences from the Los Alamos HIV sequence database. On the upper edge of the CRF01_AE group, a number of seemingly ambiguous sequences were typed as well as verified and confirmed by the HIV BLAST and RIP functions in the HIV database page, all of which were CRF01_AE.

A total of 10 different HIV-1 subtypes were identified among the 296 MSM participants (Figure 1). The predominant subtype was CRF07_BC, accounting for 57.09% (169/296), followed by CRF01_AE at 20.61% (61/296), and CRF55_01B at 5.07% (15/296); URFs accounted for 2.70% (8/296) (Table 1).

Maximum likelihood phylogenetic tree of 296 pol sequences of HIV-1 in Ningxia. A total of 61 HIV-1 sequences were branched with the CRF01_AE reference sequence (depicted in pink), 169 HIV-1 sequences were branched with the CRF07_BC reference sequence (depicted in blue), 20 HIV-1 sequences were clustered with the B reference sequence (depicted in orange), and 15 HIV-1 CRF55_01B sequences were identified (depicted in purple), 3 CRF65_cpx sequences (depicted in red), 3 CRF58_01B sequence (depicted in light red), and 8 URFs. Additionally, 43 patients were infected with other subtypes; details are provided in Figure 1.

A total of 113 cases were confirmed to have genetic resistance. Among the 296 sequenced individuals, the drug resistance prevalence was 38.18% (113/296); among the total 855 ART-treated MSM sampled, the prevalence was 13.22% (113/855). The absolute number of resistant cases increased from 9 in 2018 to 29 in 2024, but the annual sample size also increased; the Cochran-Armitage trend test showed no statistically significant trend (*p* = 0.058). Please refer to Table 1 for further information.

### Identification of HIV-1 transmission clusters

A total of 129 MSM living with HIV-1 were part of molecular transmission clusters, representing an overall clustering rate of 43.6% (129/296). Among age subgroups, individuals aged 30–39 years were the most represented in clusters, accounting for 34.1% (44/129) of clustered cases. Analysis by HIV-1 subtype revealed that individuals infected with CRF07_BC were the most likely to be part of transmission clusters. This group comprised 71.3% (92/129) of clustered individuals, However, as shown in Table 2, CRF07_BC was associated with lower odds of high-degree connectivity (AOR = 0.209), indicating that while CRF07_BC dominates large clusters, individual nodes within those clusters tend to have a lower degree.

**TABLE 2:** Factors associated with high linkage among men who have sex with men (MSM) within networks.

Characteristics	Total (*N* %)	In cluster (*N* %)	Unadjusted OR (95% CI)	*P*-value	Adjusted OR (95% CI)	*P*-value
Age						
<20	16 (5.4)	7 (5.4)	Ref	–	–	–
20∼29	93 (31.4)	36(28.0)	0.830 (0.285–2.414)	0.732	–	–
30∼39	89 (30.1)	44 (34.1)	1.044 (0.357–3.053)	0.938	–	–
40∼49	60 (20.3)	22 (17.1)	1.167 (0.383–3.556)	0.786	–	–
50∼59	29 (9.8)	17 (13.2)	0.957 (0.280–3.273)	0.944	–	–
≥60	9 (3.0)	3 (2.3)	2.722 (0.425–17.419)	0.290	–	–
Occupation					–	–
Unemployed/retiree	106 (35.8)	51 (39.5)	Ref	–	Ref	–
Farmer	41 (13.9)	16 (12.4)	1.449 (0.695–3.019)	0.322	1.895 (0.872–4.119)	0.106
Business waiter	39 (13.2)	21 (16.3)	0.795 (0.381–1.659)	0.541	0.997 (0.457–2.173)	0.994
Workers	32 (10.9)	7 (5.4)	3.577 (1.433–8.925)	0.006	4.019 (1.550–10.417)	0.004
Others and unknown	78 (26.3)	34 (26.4)	1.145 (0.634–2.069)	0.653	1.288 (0.686–2.417)	0.431
Marital status						
Married/partnered	62 (21.0)	27 (20.9)	Ref	–	–	–
Divorced/Widowed	55 (18.6)	26 (20.1)	0.86 (0.415–1.785)	0.686	–	–
Unmarried	179 (60.4)	76 (59.0)	1.045 (0.584–1.873)	0.881	–	–
Degree of education						
Illiterate	6 (2.0)	2 (1.6)	Ref		–	–
Primary school	18 (6.1)	8 (6.2)	0.625 (0.090–4.329)	0.634	–	–
Junior high school	71 (24.0)	30 (23.3)	0.683 (0.117–3.978)	0.672	–	–
High school or technical secondary school	81 (27.4)	37 (28.7)	0.595 (0.103–3.431)	0.561	–	–
College degree or above	120 (40.5)	52 (40.3)	0.654 (0.115–3.708)	0.631	–	–
Drug resistance					–	–
Yes	113 (38.2)	45 (34.9)	Ref		–	–
No	183 (61.8)	84 (65.1)	0.780 (0.485–1.255)	0.306	–	–
HIV-1 genotype						
CRF01_AE	61 (20.6)	13 (10.1)	Ref	–	Ref	–
CRF07_BC	169 (57.1)	92 (71.3)	0.227 (0.114–0.449)	<0.001	0.209 (0.104–0.420)	<0.001
CRF55_01B	15 (5.1)	8 (6.2)	0.203 (0.060–0.690)	0.011	0.201 (0.058–0.699)	0.012
URFs	8 (2.7)	1 (0.8)	1.896 (0.214–16.823)	0.566	2.041 (0.225–18.498)	0.526
Others	43 (14.5)	15 (11.6)	0.524 (0.218–1.255)	0.147	0.417 (0.169–1.028)	0.058
Place of residence						
Yinchuan city	179 (60.5)	74 (57.4)	Ref	–	–	–
Shizuishan city	29 (9.8)	9 (7.0)	1.566 (0.675–3.632)	0.296	–	–
Wuzhong city	53 (17.9)	29 (22.5)	0.583 (0.315–1.081)	0.087	–	–
Guyuan city	11 (3.7)	5 (3.9)	0.846 (0.249–2.875)	0.788	–	–
Zhongwei city	24 (8.1)	12 (9.3)	0.705 (0.300–1.655)	0.422	–	–

**FIGURE 2:**
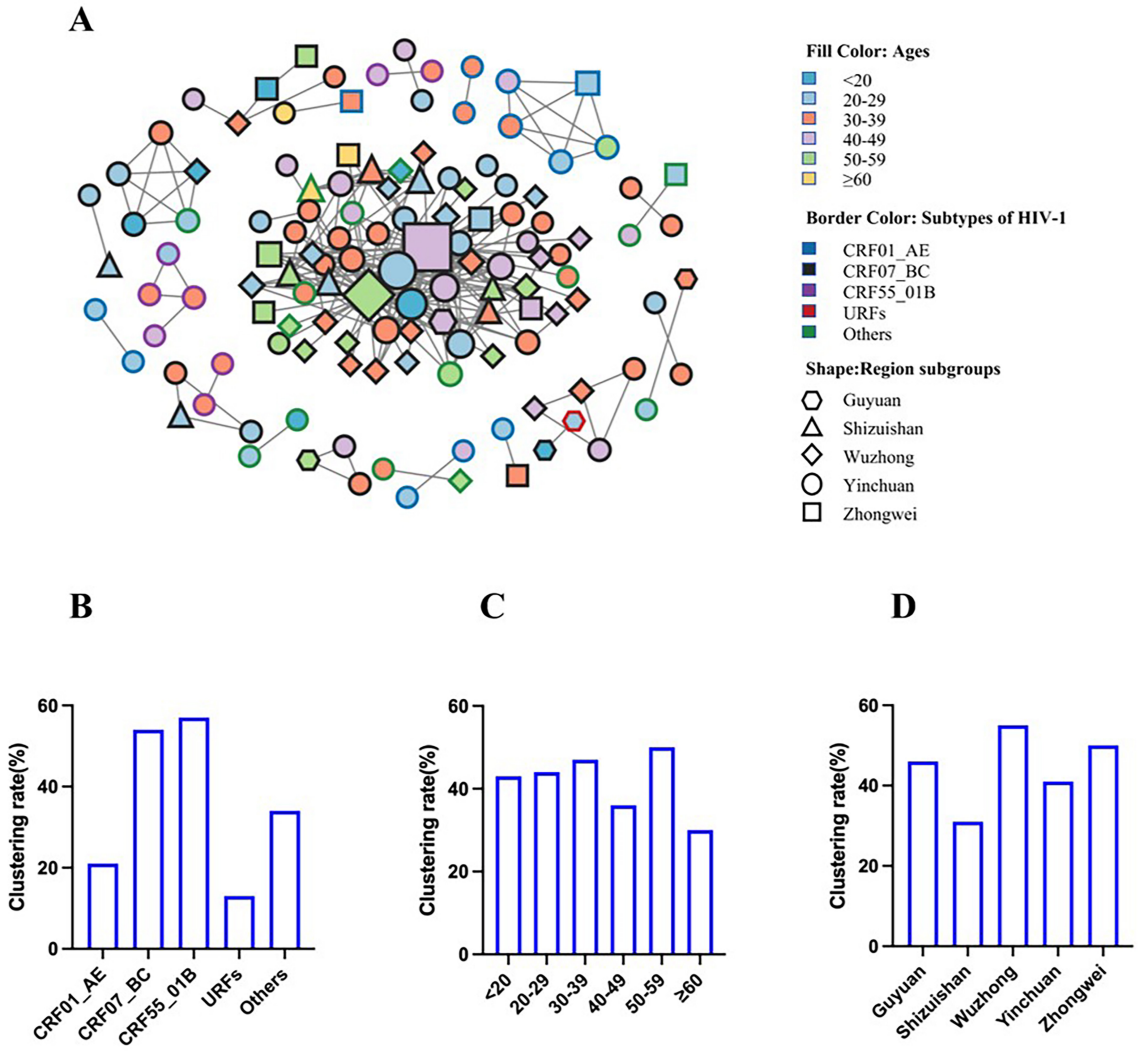
HIV-1 Transmission network among MSM in Ningxia **(A)**, the size of a node is proportional to its degree. Colors indicate different aged population groups, and different border colors indicate subtypes. Shapes indicate different region subgroups. Clustering rate among the different and subtype **(B)**, age groups **(C)**, region **(D)**.

Geographically, the majority of clustered cases were from Yinchuan, which contributed 57.4% of the total clustered MSM (Figure 2).

### Large transmission clusters

**FIGURE 3:**
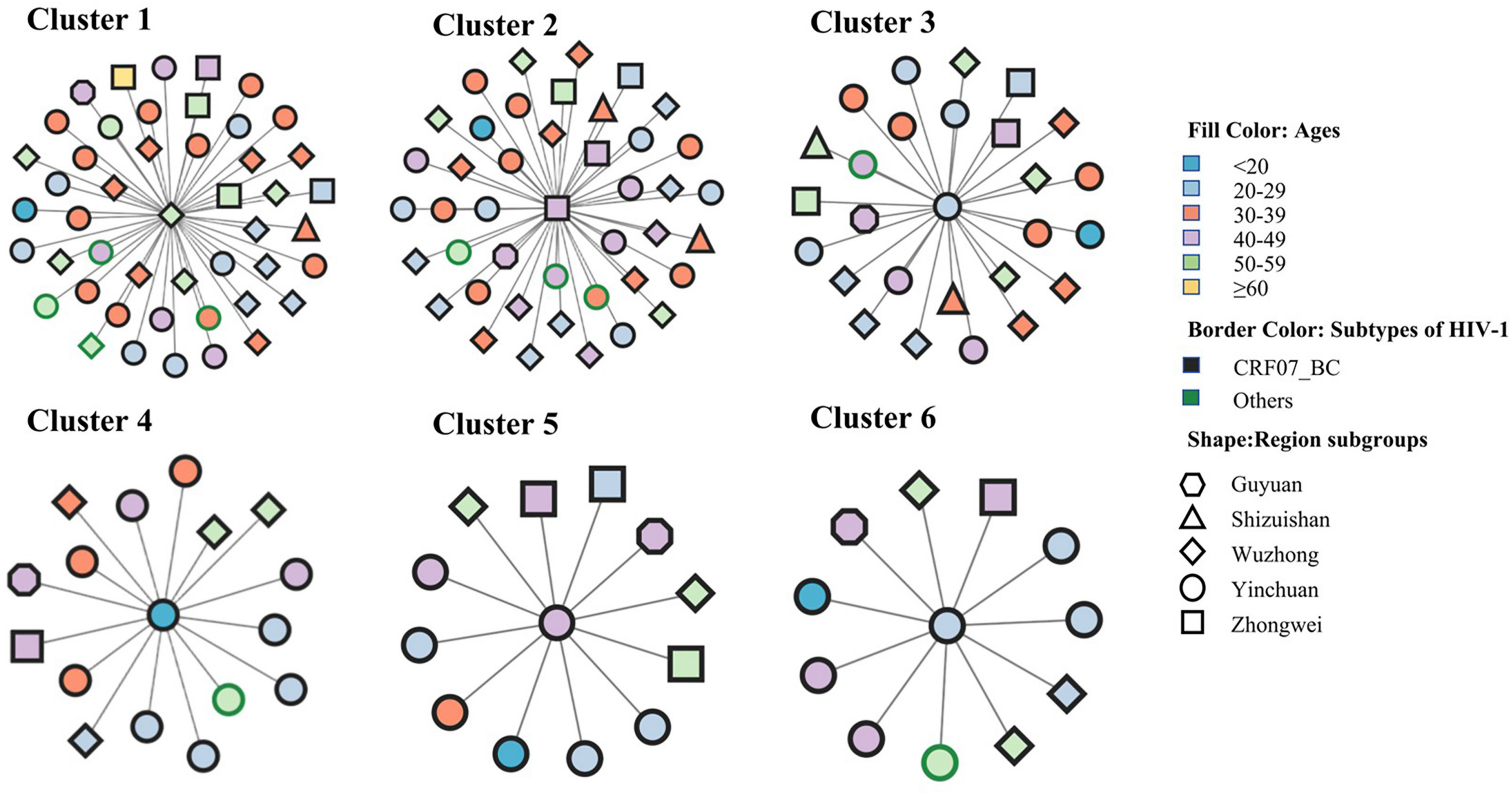
Large transmission clusters in MSM based on HIV infection stage. Nodes indicate patients. The shape represents Region city. Colours indicate different aged groups: dark blue is less than 20, light blue is 20–29, orange is 30–39, purple is 40–49, green is 50–59, yellow is more than 60. Border color represents subtypes and dark purple is CRF07_BC and green is others. Shapes indicate different region subgroups.

In accordance with the established regulations, clusters comprising more than 10 associated individuals are designated as large clusters (≥10 members). The present study identified a total of six large clusters. Figure 3 (Large transmission clusters in MSM based on HIV infection stage) illustrates the top six major transmission clusters. The largest cluster (Cluster 1) contained 47 infected individuals: 1 MSM aged < 20, 11 aged 20–29, 18 aged 30–39, and 17 aged ≥ 40. The individual identified as the highest-degree node in Cluster 1 is an MSM residing in Wuzhong (links = 47). This subject is categorized as an individual with long-term HIV-1 infection, aged 50–59 years, and characterized by the presence of the CRF07_BC subtype. In Cluster 2, the number of subjects enrolled was 43: 1 MSM aged < 20, 12 aged 20–29, 15 aged 30–39, and 15 aged ≥ 40. The geographic distribution of subjects across municipalities was as follows: 19 MSM from Yinchuan, 17 MSM from Wuzhong, 4 MSM from Zhongwei, 2 MSM from Shizuishan, and 1MSM from Guyuan. By subtype, 40 subjects belonged to CRF07_BC, while three subjects were classified as other subtypes. In Cluster 3, the number of subjects enrolled was 27, 1 MSM aged < 20, 8 MSM aged 20–29, 8 aged 30–39, and 10 MSM aged ≥ 40. The geographic distribution was as follows: 12 MSM from Yinchuan, 9 MSM from Wuzhong, 3 MSM from Zhongwei, 2 MSM from Shizuishan, and 1 MSM from Guyuan. Among the sequenced samples, 26 MSM belonged to the CRF07_BC subtype, while one was re-classified as CRF07_BC after re-sequencing and recombination analysis (the original classification as “another subtype” was due to a short recombinant fragment; all pairwise distances within the cluster were ≤1.5%.

### Transmission dynamics between regional subgroups

**FIGURE 4:**
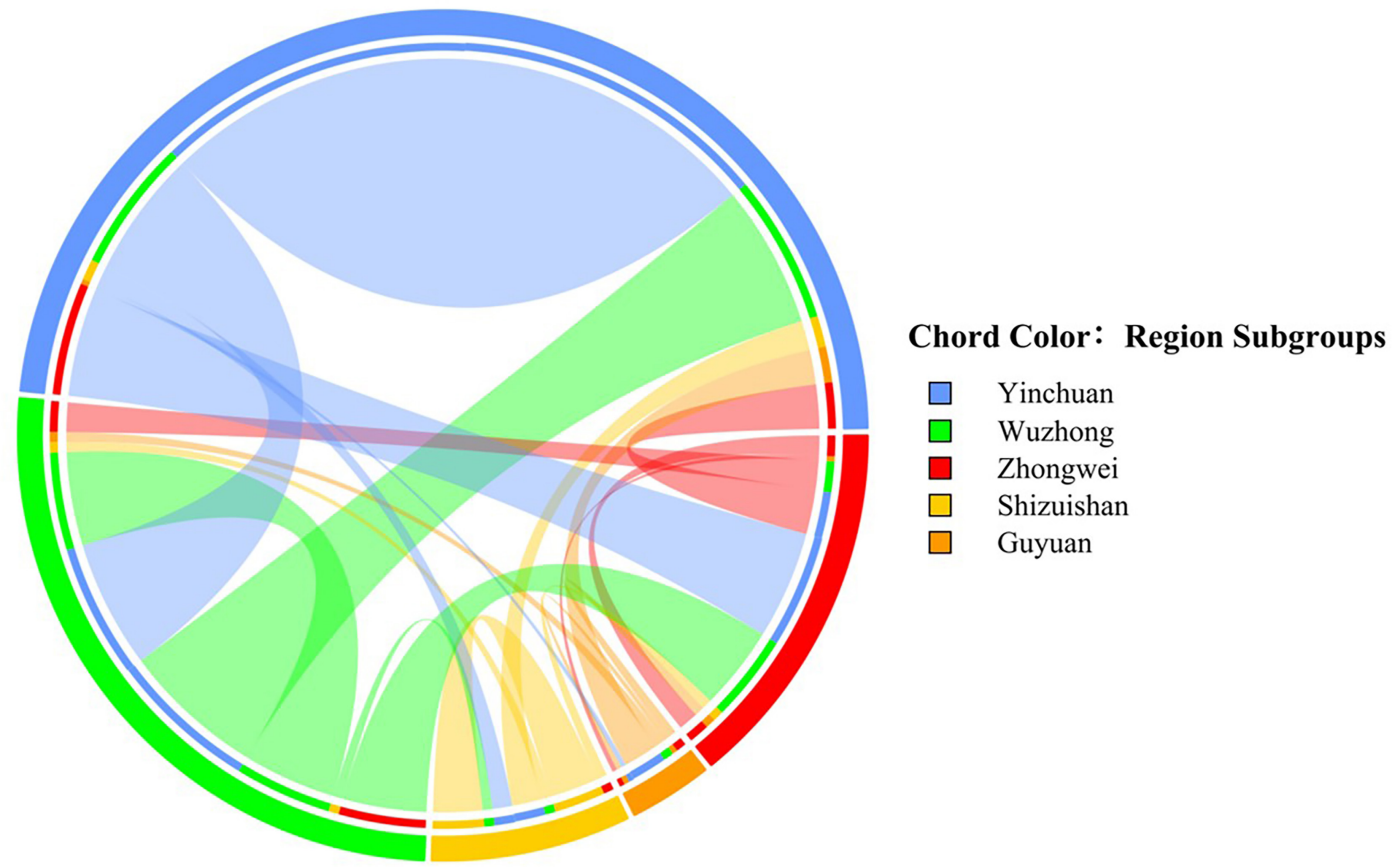
Network attribute relationships between regions. Colors indicate different region, blue represents Yinchuan city, green represents Wuzhong city, red represents Zhongwei city, yellow represents Shizuishan city and orange represents Guyuan city.

**FIGURE 5:**
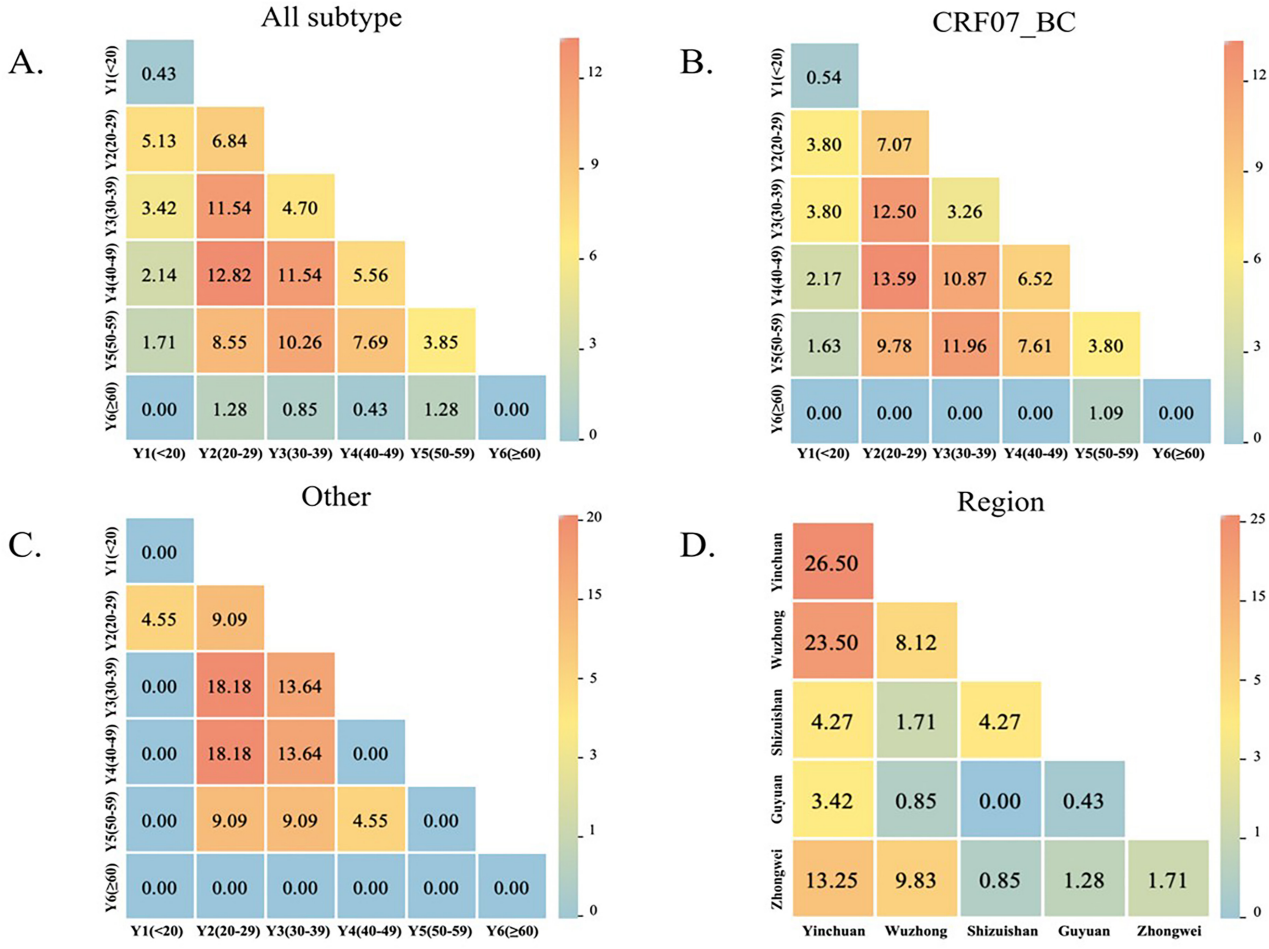
The connection intensity among all subtypes **(A)**, CRF07_BC **(B)**, others**(C)**, and region **(D)**. The connection intensity was calculated using degree (links) in the transmission network and the grid plot illustrates the connection intensity between subgroups, with higher values in the cell indicating stronger connection intensity.

Molecular network analysis reveals that MSM living with HIV-1 in Ningxia are primarily linked to younger individuals, particularly those aged 30–39 years, who accounted for a substantial proportion of total linkage connections. Regionally, the highest linkage was found in Yinchuan (34.9%) and Wuzhong (23.8%) (Figure 4). We further assessed the connection intensity of major subtypes among different age groups of MSM. The connection intensity among all subtypes (Figure 5A), CRF07_BC (Figure 5B), others (Figure 5C), and regions (Figure 5D) is shown. For all subtypes (Figure 5A), CRF07_BC (Figure 5B), and other subtypes (Figure 5C), the strongest connection intensity was among MSM aged 30–39 years. Geographically, the most robust connection intensity was within Yinchuan, followed by between Yinchuan city and Wuzhong city (Figure 5D).

**FIGURE 6:**
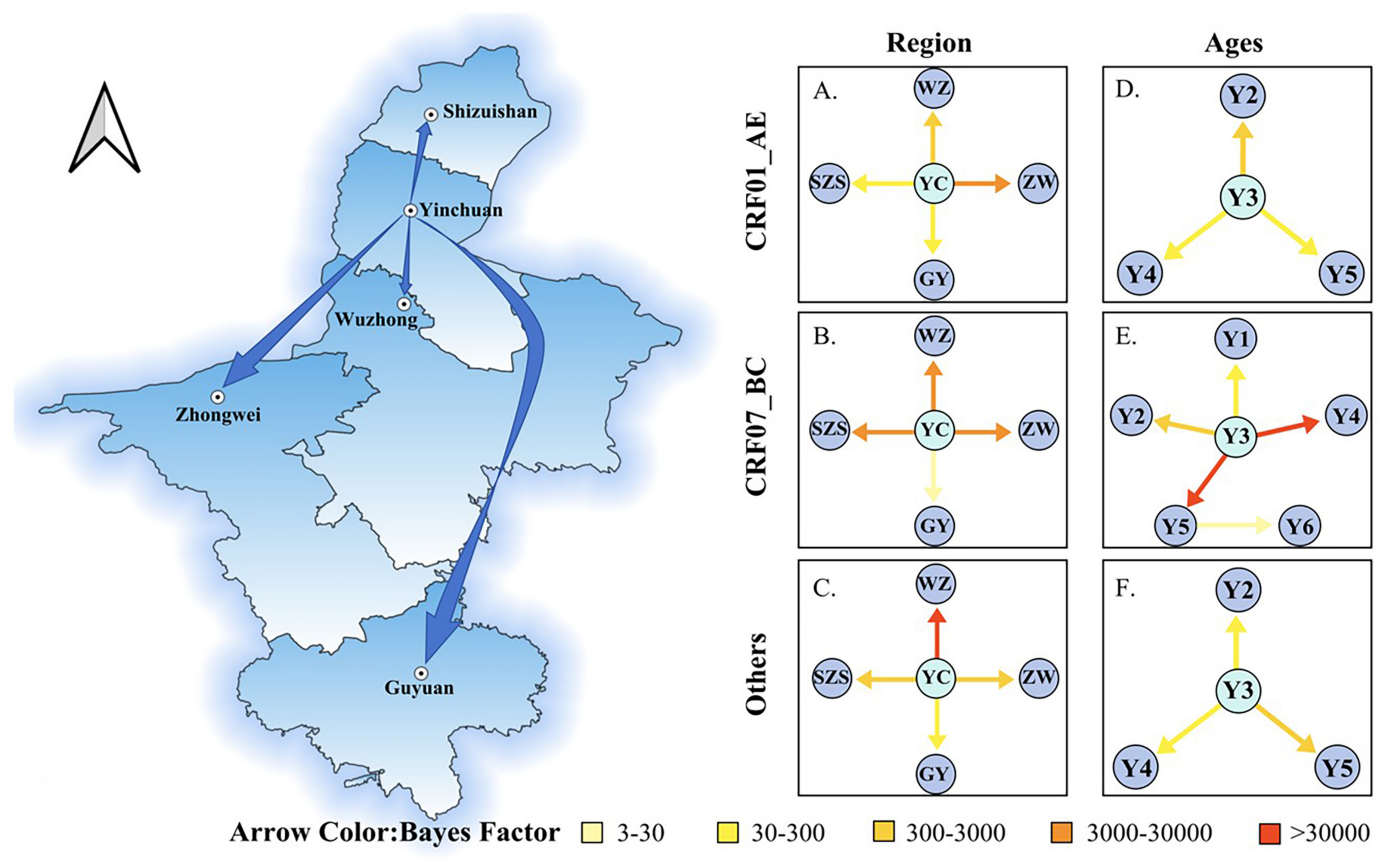
HIV-1 migration events. Presented HIV migration events among different groups in CRF01_AE **(A)**, CRF07_BC **(B)**, and others **(C)**; and different regions in CRF01_AE **(D)**, CRF07_BC **(E)**, and others **(F)**. Only results with a Bayes factor (BF) > 3 and posterior probability support = 0.8 are presented. Arrows indicate the direction of HIV-1 migration events. The colors showed the different level of BF values. Y1, MSM aged 20; Y2, MSM aged 20–29, Y3, MSM aged 30–39; Y4, MSM aged 40–49; Y5, MSM aged 50–59; Y6, MSM aged = 60. YC, Yinchuan; WZ, Wuzhong; SZS, Shizuishan; ZW, Zhongwei; GY, Guyuan.

As illustrated in Figure 6, the following data relate to HIV-1 migration events. Among all subtypes (Figure 6), CRF01_AE (Figure 6D), CRF07_BC (Figure 6E), and other subtypes (Figure 6F), the strongest migration intensity was observed in the Y3 group (MSM aged 30–39 years). Geographically, for CRF07_BC (Figure 6B) and other subtypes (Figure 6C), the most robust migration intensity was between Yinchuan city and Wuzhong city. For the CRF01_AE subtype (Figure 6A), the most robust migration intensity was between Yinchuan city and Zhongwei city.

### Comparison of R_0_ across HIV-1 subtypes in the MSM population

**FIGURE 7:**
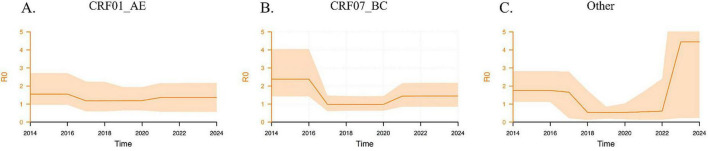
CRF01_AE **(A)**, CRF07_BC **(B)**, and others **(C)** subtypes of Ro in the MSM population.

The basic reproduction number (R_0_) of HIV-1 varies by subtype, influenced by differences in viral load dynamics, transmission fitness, and host immune responses. In MSM populations, where high-risk sexual networks and mucosal susceptibility facilitate transmission, certain subtypes may exhibit elevated R_0_ values. Our analysis revealed that the CRF01_AE genotype has remained stable in the Ningxia region, whereas the CRF07_BC genotype has shown an upward trend since 2020. Additionally, other genotypes demonstrated a substantial increase after 2022 (Figure 7: R_0_ trends for CRF01_AE, CRF07_BC, and other subtypes in the MSM population).

### Factors associated with clustering and high linkages

Multivariable logistic regression analysis was performed to identify factors associated with high-degree linkage (defined as node degree ≥ 3 connections within the network) among MSM (Table 2). Statistically significant factors:

#### Occupation

Compared to unemployed/retired individuals, workers had significantly higher odds of high-degree linkage (adjusted OR = 4.019; 95% CI: 1.550–10.417; *p* = 0.004).

#### HIV-1 subtype

Compared to CRF01_AE, both CRF07_BC (AOR = 0.209; 95% CI: 0.104–0.420; *p* < 0.001) and CRF55_01B (AOR = 0.201; 95% CI: 0.058–0.699; *p* = 0.012) were associated with lower odds of high-degree linkage. This indicates that while CRF07_BC is frequent in large clusters, individuals infected with this subtype tend to have fewer direct connections compared to those with CRF01_AE.

#### Non-significant factors (all *p* > 0.05)

Age groups, marital status, education level, drug resistance status, and place of residence showed no statistically significant association with high-degree linkage. None of the age group comparisons reached statistical significance, as indicated by confidence intervals crossing 1.0 (Table 2).

## Discussion

In recent years, molecular transmission network analysis has become a vital tool for elucidating the dynamics of HIV-1 spread and identifying key populations driving the epidemic in China. This comprehensive study provides critical insights into the molecular epidemiology, transmission dynamics, and risk factors associated with HIV-1 infection among MSM in Ningxia, China. By integrating molecular network analysis with demographic data, we have uncovered a nuanced understanding of how genetic subtypes, individual characteristics, and regional dynamics shape the HIV-1 epidemic in this setting. Our findings reveal a complex interplay between viral genetic diversity, demographic characteristics, and geographical distribution that shapes the local HIV epidemic.

The present study identified ten distinct HIV-1 subtypes. CRF07_BC (57.09%) was identified as the most prevalent, followed by CRF01_AE (20.61%) and CRF55_01B (5.07%). This distribution aligns with previous reports from China, where CRF07_BC has been widely documented in MSM populations (Zhang et al., 2025a). The predominance of CRF07_BC may reflect its enhanced transmission fitness, as suggested by its high representation in transmission clusters (71.3%). CRF07_BC does not possess an inherent predisposition toward MSM populations; rather, it has adapted to enter and thrive in this ecological niche conducive to rapid transmission, thereby becoming the primary driver of HIV-1 epidemics among MSM in China. However, our regression analysis revealed that CRF07_BC was associated with a lower individual node degree compared to CRF01_AE, suggesting that while CRF07_BC forms large clusters, the connectivity within these clusters may be more diffuse. Similar findings have been reported in other regions, such as Sichuan and Guangdong, where CRF07_BC has been associated with rapid spread among MSM (Zhang et al., 2023). Notably, CRF01_AE exhibited a stable presence, while other recombinant forms (URFs, CRF55_01B, CRF59_01B) showed increasing trends post-2022. The emergence of novel HIV recombinant strains is the result of a combination of viral evolution, host immune pressure, and epidemiological conditions. The recombination process provides a setting for what has been termed “genetic trial and error,” during which advantageous strains may arise by chance and rapidly dominate in susceptible populations and environments. This shift may indicate ongoing viral evolution and recombination, possibly driven by high-risk sexual networks. A study in Beijing also observed an uptick in URFs among MSM, underscoring the need for continuous molecular surveillance (Shen et al., 2025; Yu et al., 2025). The present study proposes the notion that continuous surveillance and functional studies are instrumental in elucidating their transmission mechanisms.

Molecular network analysis revealed that 43.6% (129/296) of sequenced individuals belonged to transmission clusters, highlighting active, localized spread. The largest cluster (Cluster 1, *n* = 47) was centered around a high-degree node (50–59 years, CRF07_BC) in Wuzhong, demonstrating how long-term infected individuals can sustain transmission chains. Furthermore, it was established that the management and control of key populations constitutes a central aspect of HIV prevention and control efforts. This aligns with studies from Europe and North America, where 5%–20% of individuals account for >80% of transmissions.

Although MSM aged 30–39 years constituted the largest proportion of clustered cases (34.1%), consistent with global data showing peak transmission in this demographic (de Jong et al., 2025), multivariable analysis did not show a statistically significant association between any age group and high-degree linkage (all 95% CIs included 1.0). Yinchuan (57.4%) and Wuzhong (23.8%) exhibited the strongest linkages, suggesting urban-driven transmission networks, possibly facilitated by dating apps and high population mobility. CRF07_BC’s over-representation in clusters (71.3%) suggests it may have a transmission advantage, as seen in prior studies (Pang et al., 2025). Our data revealed strong intercity linkages, particularly between Yinchuan and Wuzhong, indicating frequent mobility-driven transmission. This mirrors findings from other Chinese provinces where economic migration fuels HIV spread. Notably, CRF01_AE showed distinct geographical clustering (Yinchuan –Zhongwei), possibly due to different sexual network structures compared to CRF07_BC. Transmission networks among MSM map and reinforce urban networks spatially. The virus spreads by leveraging human transportation and behavioral networks. Therefore, future HIV prevention and control efforts must adopt a “spatial perspective” and “network-based approach,” effectively managing the spread of the epidemic by identifying and managing chains of transmission between cities.

National multi-center surveillance data indicate that the overall prevalence of HIV drug resistance in China remains low to moderate (approximately 3%–5%), below the WHO alert threshold of 10%. However, rising trends are observed in key populations and regions, including transmitted drug resistance (TDR) among newly diagnosed untreated individuals and acquired drug resistance (ADR) in treatment failure cases. Resistance occurs most frequently against non-nucleoside reverse transcriptase inhibitors (NNRTIs), such as efavirenz and nevirapine. The spread of resistant strains through sexual and blood-borne routes can lead to new infections that are intrinsically resistant to first-line drugs. The rate of acquired drug resistance among sequenced individuals (38.2%) exceeds the WHO alert threshold for transmitted drug resistance, although direct comparison is inappropriate as our population consists of ART-experienced, viremic individuals. But this high prevalence underscores the need for adherence support and regimen switching.

Multivariable analysis identified occupation (worker) and subtype (CRF07_BC, CRF55_01B) as significant predictors of high-degree linkage. Age was not a significant predictor. The findings of this study indicate that workers (AOR = 3.577) and farmers (AOR = 1.449) exhibited elevated clustering risks. The underlying factors contributing to the heightened HIV risk among these two groups primarily comprise structural elements, including elevated mobility, inadequate health knowledge, and constrained resources. These factors hinder the dissemination of awareness regarding protective measures and the promotion of behavioral change.

Our study reveals critical differences in the basic reproduction number (R_0_) across HIV-1 subtypes, highlighting their varying epidemic potential. Notably, CRF07_BC has emerged as a dominant driver of transmission (R_0_ > 1.5 post-2020), consistent with its predominance in transmission clusters (71.3%). This elevated R_0_ may reflect enhanced mucosal infectivity or immune evasion, as suggested by *in vitro* studies (Zhang et al., 2025b), coupled with rapid spread within dense urban MSM networks—particularly in high-turnover settings like Yinchuan (Wu et al., 2025). In contrast, CRF01_AE exhibits stable yet persistent transmission (R∼1.0–1.2), likely due to saturation in susceptible populations, mirroring trends observed in Thailand (Du et al., 2023). However, emerging recombinants (URFs/CRF55_01B) show a sharp post-2022 R_0_ increase, possibly due to founder effects from interprovincial migration and recombination advantages, such as immune evasion documented in Guangdong (Yan et al., 2025).

## Conclusion

Our study elucidates key drivers of the HIV-1 epidemic among ART-experienced, viremic MSM in Ningxia. CRF07_BC was the predominant subtype (57.09%) and appeared in large clusters, but was associated with lower individual connectivity compared to CRF01_AE. Active spread was evidenced by 43.6% of sequenced individuals forming clusters, with strong intercity linkages between Yinchuan and Wuzhong. Workers showed significantly elevated odds of high-degree linkage (AOR = 4.019). Finally, the high prevalence of acquired drug resistance (38.2% among sequenced individuals) underscores the need for adherence support and regular resistance testing.

Based on our findings, we recommend a targeted HIV prevention strategy for Ningxia’s MSM population. This involves actively targeting identified CRF07_BC transmission clusters in urban hubs like Yinchuan and Wuzhong with PrEP and enhanced testing. Interventions must be prioritized for high-risk groups, particularly individuals over 60, workers, and farmers. Establishing a regional mechanism for data-sharing and coordinated action is crucial to contain mobility-driven spread across cities. Finally, a proactive surveillance system is imperative to monitor emerging recombinant subtypes and drug resistance, guiding timely updates to treatment protocols to prevent further transmission.

## Limitations

Our study has several limitations. First, despite using an optimized genetic distance threshold, we may not have captured all active transmission chains, especially those involving closely related viral sequences due to recent transmission or limited viral evolution. Second, our inclusion criteria (ART > 6 months, VL > 200 copies/mL) introduced selection bias by selecting a subgroup enriched for virologic failure or poor adherence; the final analytical sample (*n* = 296) represented only 34.6% of the 855 ART-treated MSM sampled, so our findings apply only to ART-experienced, viremic MSM and are not generalizable to virally suppressed individuals or treatment-naïve populations. Third, the use of pol sequences, which contain drug-resistance mutations under selective pressure, may introduce convergent evolution artifacts, though our sensitivity analysis masking DRM codons suggested a 3%–5% overestimation of clustering, which we have acknowledged. Finally, while we performed sensitivity analyses across 0.5%–2.0%, no single genetic distance threshold can perfectly capture all true transmission events.

## Data Availability

The datasets presented in this article are not publicly available due to institutional policy. Requests to access the datasets should be directed to the Virology Department of Ningxia Center for Disease Control and Prevention.

## Footnotes

1
https://www.hiv.lanl.gov/

2
http://hivdb.stanford.edu
