# Supplementary material for: HIV-1 molecular transmission networks among MSM in Ningxia, China (2018–2024): insights into local transmission dynamics and drug resistance
Source: Front Microbiol. 2026 Jun 8;17:1766785. doi: 10.3389/fmicb.2026.1766785 (PMC13283995; doi:10.3389/fmicb.2026.1766785)
Supplement: Supplementary file 1 [file Presentation_1.pptx]

## Slide 1
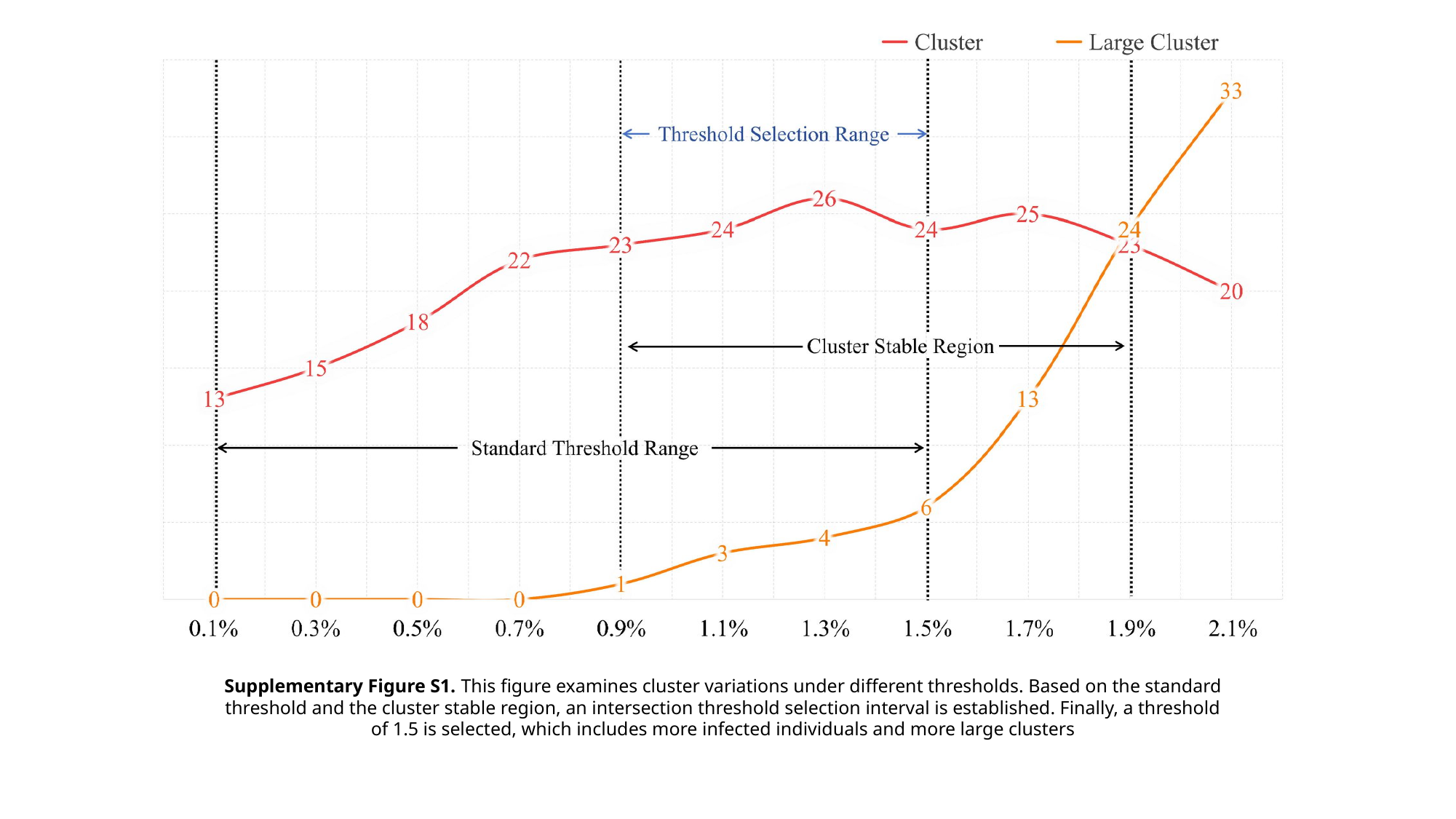

Supplementary Figure S1. This figure examines cluster variations under different thresholds. Based on the standard threshold and the cluster stable region, an intersection threshold selection interval is established. Finally, a threshold of 1.5 is selected, which includes more infected individuals and more large clusters
