# Supplementary figures and images for: HIV-1 molecular transmission networks among MSM in Ningxia, China (2018–2024): insights into local transmission dynamics and drug resistance
Source: Front Microbiol. 2026 Jun 8;17:1766785. doi: 10.3389/fmicb.2026.1766785 (PMC13283995; doi:10.3389/fmicb.2026.1766785)

## Slide 1
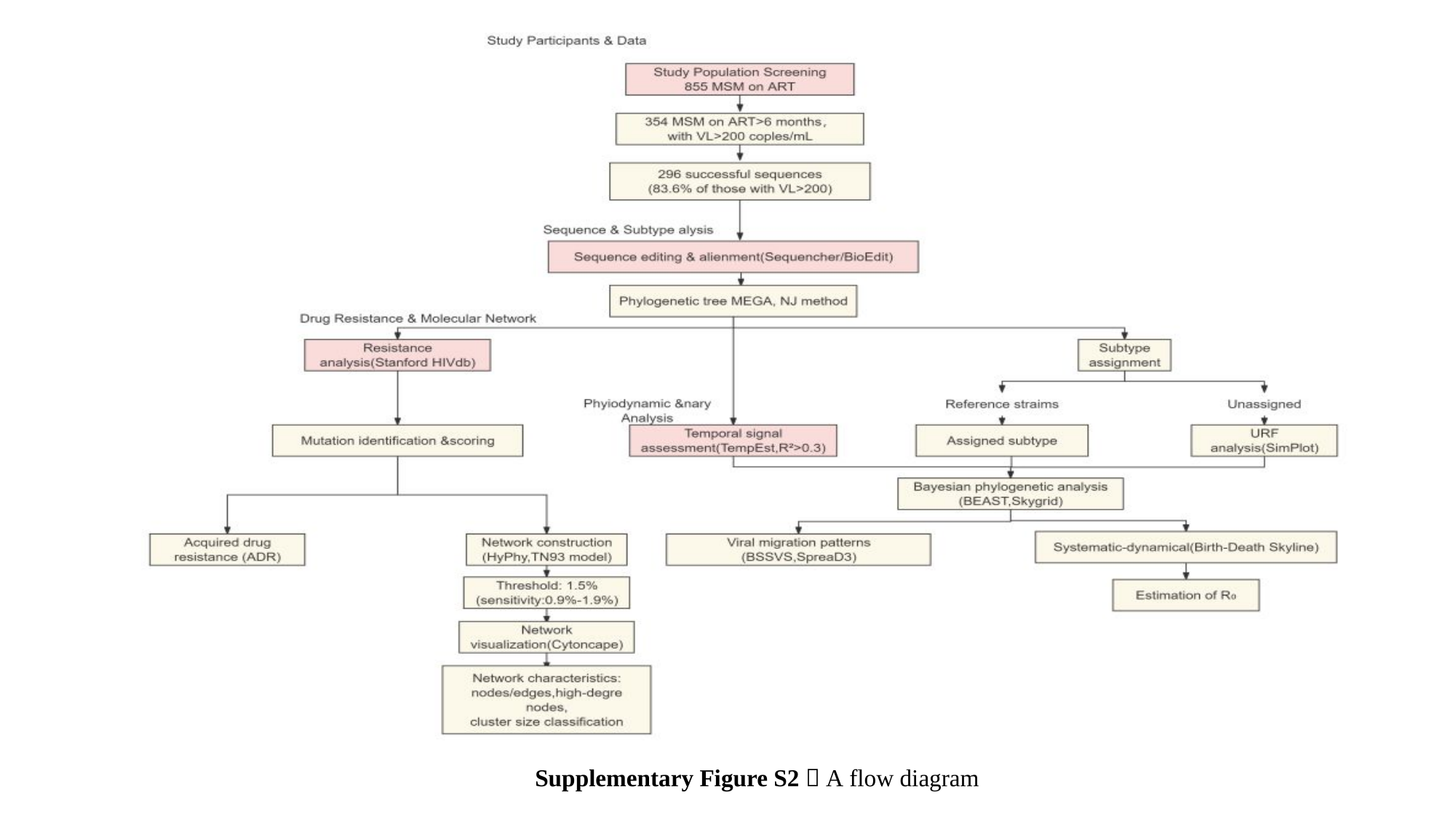

Supplementary Figure S2：A flow diagram

Supplement: Supplementary file 2 [file Presentation_2.pptx]
