# Supplementary material for: HIV-1 molecular transmission networks among MSM in Ningxia, China (2018–2024): insights into local transmission dynamics and drug resistance
Source: Front Microbiol. 2026 Jun 8;17:1766785. doi: 10.3389/fmicb.2026.1766785 (PMC13283995; doi:10.3389/fmicb.2026.1766785)
Supplement: Supplementary file 3 [file Table_1.docx]

Supplementary Table S1. The Influence of Varying Thresholds on Cluster Performance Metrics

| Thresholds(%) | 0.10 | 0.30 | 0.50 | 0.70 | 0.90 | 1.10 | 1.30 | 1.50 | 1.70 | 1.90 | 2.10 |
| --- | --- | --- | --- | --- | --- | --- | --- | --- | --- | --- | --- |
| Node | 28 | 38 | 48 | 65 | 74 | 92 | 106 | 129 | 151 | 174 | 183 |
| Edge | 16 | 29 | 39 | 58 | 72 | 111 | 154 | 234 | 320 | 482 | 647 |
| Cluster | 13 | 15 | 18 | 22 | 23 | 24 | 26 | 24 | 25 | 23 | 20 |
| Large Cluster | 0 | 0 | 0 | 0 | 1 | 3 | 4 | 6 | 13 | 24 | 33 |

Note: In this paper, a cluster composed of more than 10 nodes is referred to as a Large Cluster.
